# Supplementary material for: Comparative multiomics analysis of cell physiological state after culture in a basket bioreactor
Source: Sci Rep. 2022 Nov 23;12:20161. doi: 10.1038/s41598-022-24687-4 (PMC9686226; doi:10.1038/s41598-022-24687-4)

23666077.1:40731321:40732971:-@NW_023666077.1:40731321:40733098:-@NW_023666077.1:40734607:4073[illegible]

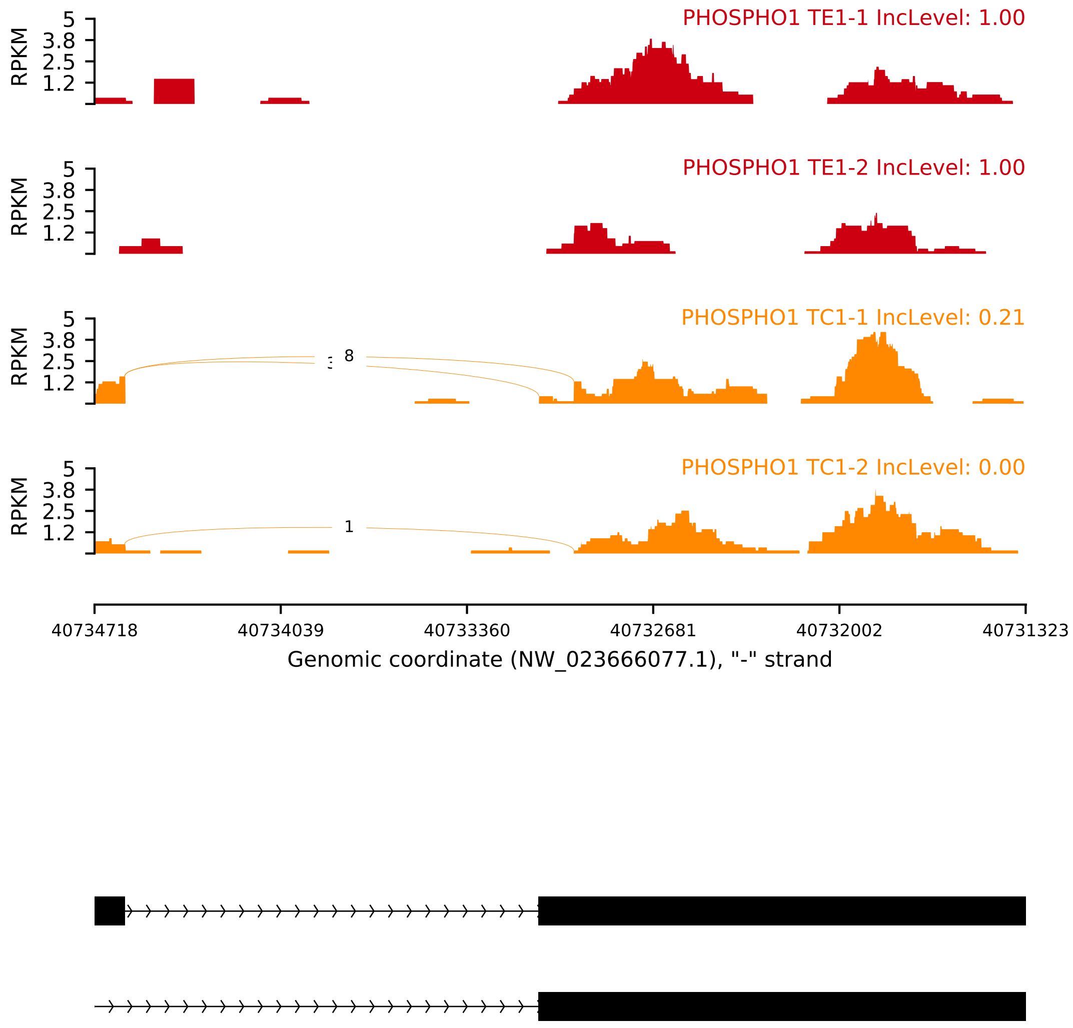

Supplement: Supplementary file 1 — Supplementary Information 1. [file 41598_2022_24687_MOESM1_ESM.zip › raw data/Transcriptomic raw data/7.AS/2.dasplot/TE1vsTC1/A3SS/10_PHOSPHO1_NW_023666077.1_40731321_40732971_-@NW_023666077.1_40731321_40733098_-@NW_023666077.1_40734607_40734717_-.pdf]
